# 3-(3,5-Dichloro­anilinocarbon­yl)propionic acid

**DOI:** 10.1107/S1600536808008556

**Published:** 2008-04-02

**Authors:** Farooq Ali Shah, M. Nawaz Tahir, Saqib Ali, Muhammad Akram Kashmiri

**Affiliations:** a Department of Chemistry, Quaid-i-Azam University, Islamabad 45320, Pakistan; b University of Sargodha, Department of Physics, Sagrodha, Pakistan; c Government College University, Department of Chemistry, Lahore, Pakistan

## Abstract

The crystal structure of the title compound, C_10_H_9_Cl_2_NO_3_, consists of dimers due to inter­molecular O—H⋯O hydrogen bonding forming an *R*_2_^2^(8) ring through the carboxyl­ groups. These dimers are linked to each other by inter­molecular hydrogen bonds between the amine group and the adjacent carbonyl O atom. A single C—Cl⋯π inter­action is also observed between the chloro-substituted aromatic rings.

## Related literature

For related literature, see: Nath *et al.* (2001 ▶); Wardell *et al.* (2006 ▶).
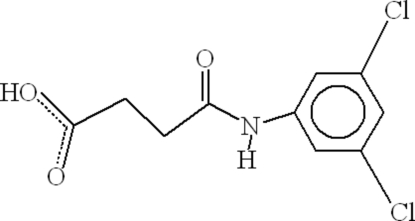


## Experimental

### 

#### Crystal data

C_10_H_9_Cl_2_NO_3_
*M* *_r_* = 262.08
Triclinic, 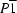

*a* = 4.8568 (2) Å
*b* = 8.6677 (4) Å
*c* = 13.9038 (8) Å
α = 74.467 (3)°
β = 80.495 (2)°
γ = 82.712 (3)°
*V* = 554.09 (5) Å^3^
*Z* = 2
Mo *K*α radiation
μ = 0.57 mm^−1^
*T* = 296 (2) K
0.25 × 0.12 × 0.10 mm

#### Data collection

Bruker Kappa APEXII CCD diffractometer
Absorption correction: multi-scan (*SADABS*; Bruker, 2005 ▶) *T*_min_ = 0.870, *T*_max_ = 0.945
12157 measured reflections
2971 independent reflections
2065 reflections with *I* > 2σ(*I*)
*R*_int_ = 0.026

#### Refinement

*R*[*F* ^2^ > 2σ(*F* ^2^)] = 0.040
*wR*(*F* ^2^) = 0.125
*S* = 1.07
2971 reflections
172 parameters
Only H-atom coordinates refined
Δρ_max_ = 0.27 e Å^−3^
Δρ_min_ = −0.43 e Å^−3^

### 

Data collection: *APEX2* (Bruker, 2007 ▶); cell refinement: *APEX2*; data reduction: *SAINT* (Bruker, 2007 ▶); program(s) used to solve structure: *SHELXS97* (Sheldrick, 2008 ▶); program(s) used to refine structure: *SHELXL97* (Sheldrick, 2008 ▶); molecular graphics: *ORTEP-3 for Windows* (Farrugia, 1997 ▶ ▶) and *PLATON* (Spek, 2003 ▶); software used to prepare material for publication: *WinGX* (Farrugia, 1999) and *PLATON*.

## Supplementary Material

Crystal structure: contains datablocks global, I. DOI: 10.1107/S1600536808008556/fj2109sup1.cif

Structure factors: contains datablocks I. DOI: 10.1107/S1600536808008556/fj2109Isup2.hkl

Additional supplementary materials:  crystallographic information; 3D view; checkCIF report

## Figures and Tables

**Table 1:** Hydrogen-bond geometry (Å, °)

*D*—H⋯*A*	*D*—H	H⋯*A*	*D*⋯*A*	*D*—H⋯*A*
N1—H1*A*⋯O3^i^	0.84 (3)	2.07 (3)	2.904 (2)	175 (2)
O1—H1⋯O2^ii^	0.92 (4)	1.74 (4)	2.658 (3)	175 (4)
C7—Cl1⋯*Cg*^iii^	1.74 (1)	3.54 (1)	4.033 (2)	93 (1)

Symmetry codes: (i) 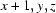; (ii) 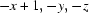; (iii) 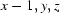. *Cg* is the centroid of atoms C5–C10.

**Fig. 1.**
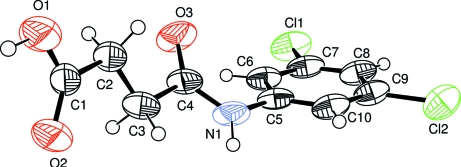
ORTEP-3 for Windows (Farrugia, 1997) drawing of the title compound, C10H9Cl2NO3 with the atom numbering scheme. The thermal ellipsoids are drawn at the 50% probability level. H-atoms are shown by small circles of arbitrary radii.

**Fig. 2.**
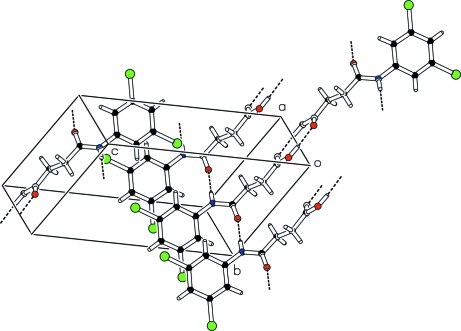
The unit cell packing of (I) (Spek, 2003), showing the dimeric nature and the linkage of dimers.

## supplementary crystallographic information

### Comment

Carboxylic acids catch the interest of people due to wide use of their metal complexes in biological and industrial field. On the other hand amino acids are one of the best sources to formulate the structure-activity correlation of metal derivatives as a biologically active agent (Nath *et al.*, 2001) and widen the scope of investigation on the coordination behavior of the ligand in biological system. The title compound (I) has been prepared for complexation with different metals.

The structure of 3-(3-Nitrophenylaminocarbonyl)-propionic acid (Wardell *et al.*, 2006) has been published. The title compound have replacement of 3-nitro with Cl and also an additional Cl-atom at 5-position of benzene ring. Therefore, the bond distances and packing of (I) is being compared with the mentioned reported structure. In (I) the C==O bond distances for carboxylate and carbonyl group have values of (C1==O2: 1.219 (3) Å) and (C4==O3: 1.225 (2) Å) in comparison to 1.223 (2) and 1.2214 (17) Å, respectively. The C—N bond distances are compareable within experimental errors. In both compounds similar intermolecular H-bonding (Table 1, Fig. 2) has been observed. The dihedral angle between the aromatic ring A(C5—C10) and (C1,C2,C3,O1,O2) have a value of 82.24 (8)°, whereas with (N1,C3,C4,O3) its value is 44.42 (12)°. The value of dihedral angle between (C1,C2,C3,O1,O2) and (N1,C3,C4,O3) is 38.36 (13)°. There exist a single C—Cl···π interaction at a distance of 3.5398 (11) Å [C7—CL1···CgA^iii^: symmetry code iii = -1 + *x*, *y*, *z*].

### Experimental

3,5-Dichloroaniline (16.2 g, 0.1 mole) and succinic anhydride (10 g, 0.1 mole) were dissolved in glacial acetic acid separately and mixed. The mixed solution was stirred at room temperature for 24 h. The precipitated material was filtered, washed with distilled water and dried at 413–423 K. The title compound (I) was obtained by recrystallizing the dried product using aceton. (Yield: 90%, m.p. 437 K).

### Crystal data

**Table:**

C_10_H_9_Cl_2_NO_3_	*Z* = 2
*M**_r_* = 262.08	*F*_000_ = 268
Triclinic, *P*1	*D*_x_ = 1.571 Mg m^−^^3^
Hall symbol: -P 1	Mo *K*α radiation λ = 0.71073 Å
*a* = 4.8568 (2) Å	Cell parameters from 2971 reflections
*b* = 8.6677 (4) Å	θ = 1.5–29.2º
*c* = 13.9038 (8) Å	µ = 0.58 mm^−^^1^
α = 74.467 (3)º	*T* = 296 (2) K
β = 80.495 (2)º	Needle, colourless
γ = 82.712 (3)º	0.25 × 0.12 × 0.10 mm
*V* = 554.09 (5) Å^3^	

### Data collection

**Table:**

Bruker KappaAPEXII CCD diffractometer	2971 independent reflections
Radiation source: fine-focus sealed tube	2065 reflections with *I* > 2σ(*I*)
Monochromator: graphite	*R*_int_ = 0.027
Detector resolution: 7.4 pixels mm^-1^	θ_max_ = 29.2º
*T* = 296(2) K	θ_min_ = 1.5º
ω scans	*h* = −6→6
Absorption correction: multi-scan(SADABS; Bruker, 2005)	*k* = −11→11
*T*_min_ = 0.870, *T*_max_ = 0.945	*l* = −19→18
12157 measured reflections	

### Refinement

**Table:**

Refinement on *F*^2^	Secondary atom site location: difference Fourier map
Least-squares matrix: full	Hydrogen site location: inferred from neighbouring sites
*R*[*F*^2^ > 2σ(*F*^2^)] = 0.040	Only H-atom coordinates refined
*wR*(*F*^2^) = 0.125	*w* = 1/[σ^2^(*F*_o_^2^) + (0.0492*P*)^2^ + 0.2158*P*] where *P* = (*F*_o_^2^ + 2*F*_c_^2^)/3
*S* = 1.07	(Δ/σ)_max_ = 0.001
2971 reflections	Δρ_max_ = 0.27 e Å^−^^3^
172 parameters	Δρ_min_ = −0.43 e Å^−^^3^
Primary atom site location: structure-invariant direct methods	Extinction correction: none

### Special details

**Table:**

Geometry. All e.s.d.'s (except the e.s.d. in the dihedral angle between two l.s. planes) are estimated using the full covariance matrix. The cell e.s.d.'s are taken into account individually in the estimation of e.s.d.'s in distances, angles and torsion angles; correlations between e.s.d.'s in cell parameters are only used when they are defined by crystal symmetry. An approximate (isotropic) treatment of cell e.s.d.'s is used for estimating e.s.d.'s involving l.s. planes.
Refinement. Refinement of *F*^2^ against ALL reflections. The weighted *R*-factor *wR* and goodness of fit *S* are based on *F*^2^, conventional *R*-factors *R* are based on *F*, with *F* set to zero for negative *F*^2^. The threshold expression of *F*^2^ > σ(*F*^2^) is used only for calculating *R*-factors(gt) *etc*. and is not relevant to the choice of reflections for refinement. *R*-factors based on *F*^2^ are statistically about twice as large as those based on *F*, and *R*- factors based on ALL data will be even larger.

### Fractional atomic coordinates and isotropic or equivalent isotropic displacement parameters (Å^2^)

**Table:**

	*x*	*y*	*z*	*U*_iso_*/*U*_eq_	
Cl1	−0.50817 (12)	1.03658 (7)	0.31192 (6)	0.0754 (2)	
Cl2	0.18783 (13)	0.70434 (7)	0.57110 (5)	0.0704 (2)	
O1	0.1715 (4)	0.0213 (3)	0.07439 (17)	0.0910 (7)	
H1	0.281 (8)	−0.046 (4)	0.039 (3)	0.109*	
O2	0.5114 (3)	0.1840 (3)	0.01798 (15)	0.0829 (6)	
O3	−0.1931 (3)	0.5285 (3)	0.18654 (16)	0.0814 (6)	
N1	0.2023 (3)	0.5827 (3)	0.23147 (16)	0.0607 (5)	
H1A	0.377 (6)	0.563 (3)	0.222 (2)	0.073*	
C1	0.2819 (4)	0.1536 (4)	0.06543 (17)	0.0629 (7)	
C2	0.1014 (4)	0.2656 (4)	0.1188 (2)	0.0636 (7)	
H2A	−0.054 (6)	0.295 (3)	0.089 (2)	0.076*	
H2B	0.031 (6)	0.202 (3)	0.186 (2)	0.076*	
C3	0.2413 (4)	0.4095 (4)	0.1194 (2)	0.0653 (7)	
H3A	0.413 (6)	0.381 (3)	0.139 (2)	0.078*	
H3B	0.275 (6)	0.480 (3)	0.051 (2)	0.078*	
C4	0.0626 (4)	0.5118 (3)	0.18182 (18)	0.0588 (6)	
C5	0.0789 (4)	0.6763 (3)	0.29919 (18)	0.0525 (5)	
C6	−0.1369 (4)	0.7959 (3)	0.2744 (2)	0.0558 (5)	
H6	−0.196 (5)	0.813 (3)	0.208 (2)	0.067*	
C7	−0.2454 (4)	0.8838 (2)	0.3433 (2)	0.0556 (6)	
C8	−0.1529 (4)	0.8582 (2)	0.4348 (2)	0.0565 (6)	
H8	−0.234 (6)	0.918 (3)	0.4805 (19)	0.068*	
C9	0.0627 (4)	0.7390 (2)	0.45673 (19)	0.0529 (5)	
C10	0.1799 (4)	0.6482 (2)	0.39000 (19)	0.0534 (5)	
H10	0.323 (5)	0.569 (3)	0.4041 (18)	0.064*	

### Atomic displacement parameters (Å^2^)

**Table:**

	*U*^11^	*U*^22^	*U*^33^	*U*^12^	*U*^13^	*U*^23^
Cl1	0.0467 (3)	0.0511 (3)	0.1229 (6)	0.0104 (2)	−0.0224 (3)	−0.0129 (3)
Cl2	0.0688 (4)	0.0548 (3)	0.0942 (5)	−0.0021 (3)	−0.0275 (3)	−0.0220 (3)
O1	0.0561 (10)	0.1287 (19)	0.1042 (16)	−0.0241 (11)	0.0243 (10)	−0.0704 (14)
O2	0.0471 (9)	0.1186 (16)	0.0902 (13)	−0.0130 (9)	0.0204 (9)	−0.0543 (12)
O3	0.0227 (6)	0.1166 (16)	0.1185 (15)	0.0053 (8)	−0.0064 (8)	−0.0601 (13)
N1	0.0212 (7)	0.0772 (13)	0.0841 (14)	0.0006 (7)	−0.0002 (7)	−0.0273 (11)
C1	0.0344 (9)	0.110 (2)	0.0533 (13)	−0.0055 (11)	−0.0032 (9)	−0.0381 (13)
C2	0.0293 (9)	0.104 (2)	0.0631 (15)	−0.0026 (10)	−0.0004 (9)	−0.0360 (14)
C3	0.0273 (9)	0.0925 (19)	0.0749 (16)	0.0001 (10)	0.0053 (9)	−0.0290 (14)
C4	0.0240 (8)	0.0786 (15)	0.0716 (15)	−0.0001 (8)	−0.0007 (8)	−0.0208 (12)
C5	0.0242 (7)	0.0512 (11)	0.0789 (15)	−0.0053 (7)	0.0001 (8)	−0.0146 (10)
C6	0.0306 (8)	0.0564 (13)	0.0747 (15)	−0.0046 (8)	−0.0058 (9)	−0.0074 (11)
C7	0.0301 (8)	0.0381 (10)	0.0934 (17)	−0.0015 (7)	−0.0078 (9)	−0.0086 (10)
C8	0.0404 (10)	0.0379 (11)	0.0926 (18)	−0.0046 (8)	−0.0083 (10)	−0.0187 (11)
C9	0.0406 (9)	0.0367 (10)	0.0822 (15)	−0.0072 (8)	−0.0133 (9)	−0.0118 (10)
C10	0.0337 (9)	0.0388 (10)	0.0864 (17)	−0.0017 (7)	−0.0114 (9)	−0.0125 (10)

### Geometric parameters (Å, °)

**Table:**

Cl1—C7	1.737 (2)	C3—C4	1.503 (3)
Cl2—C9	1.734 (2)	C3—H3A	0.91 (3)
O1—C1	1.295 (3)	C3—H3B	0.99 (3)
O1—H1	0.92 (4)	C5—C10	1.380 (3)
O2—C1	1.219 (3)	C5—C6	1.394 (3)
O3—C4	1.225 (2)	C6—C7	1.378 (3)
N1—C4	1.343 (3)	C6—H6	0.98 (3)
N1—C5	1.415 (3)	C7—C8	1.372 (3)
N1—H1A	0.84 (3)	C8—C9	1.386 (3)
C1—C2	1.488 (3)	C8—H8	0.93 (3)
C2—C3	1.497 (4)	C9—C10	1.381 (3)
C2—H2A	0.90 (3)	C10—H10	0.92 (3)
C2—H2B	0.97 (3)		
			
C1—O1—H1	113 (2)	O3—C4—C3	121.8 (2)
C4—N1—C5	125.67 (16)	N1—C4—C3	115.51 (17)
C4—N1—H1A	114.4 (19)	C10—C5—C6	120.6 (2)
C5—N1—H1A	119.8 (19)	C10—C5—N1	118.47 (19)
O2—C1—O1	123.4 (2)	C6—C5—N1	121.0 (2)
O2—C1—C2	123.1 (3)	C7—C6—C5	118.0 (2)
O1—C1—C2	113.5 (2)	C7—C6—H6	124.9 (15)
C1—C2—C3	113.79 (18)	C5—C6—H6	117.1 (15)
C1—C2—H2A	107.1 (18)	C8—C7—C6	123.18 (19)
C3—C2—H2A	111.1 (18)	C8—C7—Cl1	118.42 (18)
C1—C2—H2B	107.3 (16)	C6—C7—Cl1	118.39 (19)
C3—C2—H2B	114.0 (16)	C7—C8—C9	117.3 (2)
H2A—C2—H2B	103 (2)	C7—C8—H8	121.1 (17)
C2—C3—C4	112.22 (18)	C9—C8—H8	121.6 (17)
C2—C3—H3A	111.7 (18)	C10—C9—C8	121.8 (2)
C4—C3—H3A	111.0 (17)	C10—C9—Cl2	119.54 (16)
C2—C3—H3B	110.6 (17)	C8—C9—Cl2	118.68 (19)
C4—C3—H3B	105.7 (17)	C5—C10—C9	119.2 (2)
H3A—C3—H3B	105 (2)	C5—C10—H10	118.7 (16)
O3—C4—N1	122.7 (2)	C9—C10—H10	122.1 (16)
			
O2—C1—C2—C3	−7.0 (4)	C5—C6—C7—C8	−0.6 (3)
O1—C1—C2—C3	172.9 (2)	C5—C6—C7—Cl1	178.35 (15)
C1—C2—C3—C4	−174.8 (2)	C6—C7—C8—C9	0.9 (3)
C5—N1—C4—O3	4.0 (4)	Cl1—C7—C8—C9	−178.06 (14)
C5—N1—C4—C3	−176.1 (2)	C7—C8—C9—C10	−0.4 (3)
C2—C3—C4—O3	−34.9 (4)	C7—C8—C9—Cl2	179.37 (15)
C2—C3—C4—N1	145.1 (2)	C6—C5—C10—C9	0.6 (3)
C4—N1—C5—C10	133.9 (2)	N1—C5—C10—C9	179.70 (17)
C4—N1—C5—C6	−47.0 (3)	C8—C9—C10—C5	−0.3 (3)
C10—C5—C6—C7	−0.2 (3)	Cl2—C9—C10—C5	179.91 (15)
N1—C5—C6—C7	−179.26 (18)		

### Hydrogen-bond geometry (Å, °)

**Table:**

*D*—H···*A*	*D*—H	H···*A*	*D*···*A*	*D*—H···*A*
N1—H1A···O3^i^	0.84 (3)	2.07 (3)	2.904 (2)	175 (2)
O1—H1···O2^ii^	0.92 (4)	1.74 (4)	2.658 (3)	175 (4)
C7—Cl1···Cg^iii^	1.737 (2)	3.5398 (11)	4.033 (2)	93.34 (7)

Symmetry codes: (i) *x*+1, *y*, *z*; (ii) −*x*+1, −*y*, −*z*; (iii) *x*−1, *y*, *z*.
